# “I just felt there was not going to be issues” exploring local definitions of exclusive breastfeeding and adequate complementary feeding within communities in Jigawa state, Nigeria

**DOI:** 10.1038/s41598-026-41749-z

**Published:** 2026-02-26

**Authors:** Funmilayo Shittu, Carina King, Ayobami A. Bakare, Damola Bakare, Julius Salako, Agnese Iuliano, Susanne Rautiainen, Tim Colbourn, Rochelle A. Burgess, Adegoke G. Falade

**Affiliations:** 1 Department of Paediatrics, University of Ibadan, Ibadan, Nigeria; 2 Department of Global Public Health, Karolinska Institutet, Stockholm, Sweden; 3 Department of Community Medicine, University of Ibadan, Ibadan, Nigeria; 4 Clinical Epidemiology Division, Department of Medicine, Solna, Karolinska Institutet, Stockholm, Sweden; 5 Institute for Global Health, University College London, London, UK

**Keywords:** Infant, Exclusive breastfeeding, Complementary feeding, Nigeria, Anthropology, Health care

## Abstract

Exclusive breastfeeding (EBF) and appropriate complementary feeding (CF) are important for child survival and development. While global guidelines provide clear definitions for these practices, interpretations within communities can differ, influencing infant feeding behaviours. This study explored how EBF and CF are understood, and how these understandings shape infant feeding and nutrition within a community setting in Jigawa State, northern Nigeria. We conducted a qualitative study using life-history interviews and household observations within an ethnographic process evaluation in Kiyawa LGA, Jigawa State, Nigeria, between July 2020 and November 2022. The data formed part of the INSPIRING Jigawa cluster randomized controlled trial process evaluation. From 90 women recruited for the ethnography, we purposively selected 36 women aged 16–49 years who had breastfed in the preceding two years. Data was analysed from midline interviews and notes from monthly facility and household observations. Interviews were conducted in Hausa, transcribed, translated into English, and analysed using reflexive thematic analysis. We found that while most mothers could state the correct definition of EBF as feeding only breastmilk for the first six months, their interpretation allowed for the early introduction of water, and other water solutions without perceiving this as a breach of EBF. Complementary feeding was similarly defined based on infant cues and cultural beliefs, with many mothers introducing family foods before six months in response to perceived signs of hunger or under the influence of older family members. These locally adapted definitions reflected deep-rooted cultural norms and caregiving traditions. While communities in Jigawa are aware of global definitions for EBF, these are not commonly adopted or practiced. Interventions aiming to improve infant feeding practices must consider culturally embedded beliefs around the provision of water earlier than recommended, and engage influential family and community members to align local practices with recommended guidelines.

**Supplementary Information:**

The online version contains supplementary material available at 10.1038/s41598-026-41749-z.

## Introduction

Optimal infant and young child feeding practices, particularly exclusive breastfeeding (EBF) and complementary feeding (CF), are widely recognised as critical for child survival, growth, and development^1^. The World Health Organization (WHO) defines EBF as feeding an infant only breast milk for the first six months of life, without any additional food or drink, not even water, except for oral rehydration solution, or drops/syrups of vitamins, minerals, or medicines when medically indicated^1^. CF refers to the timely introduction of solid, semi-solid, or soft foods at six months of age, alongside continued breastfeeding, to meet the growing nutritional requirements of the child^2^. Adequate CF is characterised by dietary diversity, meal frequency, and appropriate meal quantity, contributing to a child’s optimal growth and development^3^. Despite widespread promotion of these guidelines, there is a large gap between international recommendations and coverage^1^.

Several studies have documented how sociocultural norms, household power relations, and local beliefs influence how these practices are understood and implemented^4–6^. In northern Nigeria, EBF is often misunderstood. Although mothers report practicing EBF, qualitative studies have reported provision of water, herbal concoctions, or gripe water being given to infants, especially during hot weather, periods of perceived illness, or infant discomfort^6,7^. These practices were explained by the belief that breast milk alone is insufficient to quench a child’s thirst or provide protection against illness in challenging environmental conditions^6^. Other studies from northern Nigeria indicate that caregivers perceive breast milk to be ‘hot’ or ‘light’ and incapable of fully satisfying a child, particularly during the hot dry season^4,8^. This perception often leads to the early introduction of water or traditional herbal preparations within the first months of life. Additionally, the advice of elder female relatives, particularly grandmothers, significantly influences maternal decisions about infant feeding. Grandmothers are often the custodians of traditional knowledge and cultural practices, and their support or disapproval can determine whether EBF is practiced as recommended in this setting^9–11^.

Similar divergences between global recommendations and local understandings are evident in the case of complementary feeding practices in Northern Nigeria. While biomedical guidelines emphasise diverse, nutrient-rich complementary foods, some studies from Nigeria report complementary-feeding practices and perceptions that prioritise food quantity or early introduction of staple or cereal-based complementary foods, focusing on satiety or perceived sufficiency rather than dietary diversity or nutritional content^12,13^. Studies have shown that CF typically involves the early introduction of staples (e.g. pap), but little addition of protein-rich or micronutrient-dense foods due to limited availability, poverty, and cultural beliefs about appropriate infant foods^14,15^. Decisions regarding what and when to feed a child are also` influenced by household hierarchies, with male heads of households controlling food purchases and elder women determining feeding norms^16,17^.

Although several studies have explored infant feeding practices in northern Nigeria, most have focused on the prevalence and determinants of EBF and CF, without explicitly examining how these concepts are defined and understood within communities^4,7,18^. A 2016 systematic review on mother’s understanding of EBF identified 4 qualitative studies exploring this topic, which highlighted inaccurate understandings of EBF, including commonly believing that giving water or traditional remedies was still acceptable. Cultural norms, family influence, and unclear health messaging shaped these misconceptions, highlighting the need for clearer, context-specific communication on EBF, however no studies were found from Nigeria^19^. Given the high burden of malnutrition and child mortality in Northern Nigeria, understanding local definitions of EBF and CF are essential for identifying culturally embedded barriers to optimal infant feeding practices and designing interventions that are both acceptable and effective within these communities. We therefore aimed to explore how community definitions of exclusive breastfeeding (EBF) and complementary feeding (CF) in Jigawa State, Northern Nigeria, shape infant feeding practices and child nutrition outcomes.

## Methods

### Study design

We conducted a qualitative study using life-history interviews with women of childbearing age (16–49 years old) and both facility and household observations conducted as part of an ethnographic process evaluation conducted between July 2020 and November 2022. We followed the Consolidated Criteria for Reporting Qualitative Research (COREQ) to ensure comprehensive and transparent reporting of the study methods and findings^20^. The study was set in Kiyawa Local Government Area (LGA), Jigawa State, Nigeria, and was part of the INSPIRING Jigawa cluster randomized controlled trial process evaluation (ISRCTN3921355, registered: 11th December 2019)^21,22^. The INSPIRING trial implemented a whole-systems strengthening intervention to reduce under-five mortality, particularly focusing on pneumonia, through participatory learning and action, community-healthcare accountability, and health system strengthening. 

In our previous study exploring household power in EBF practices, the importance of water in feeding practices emerged as a sub-theme under the beliefs and cultural norms within households that impact EBF^6^. Given the limited literature in this area, and the unexpectedly high self-reporting of EBF^23^, we decided to re-analyse these data to explore in more depth how water is understood as part of the local definition of EBF, as well as exploring definitions of adequate CF and how these link to the nutritional status of children in this setting.

### Setting

This study was conducted in Kiyawa LGA of Jigawa State, with an estimated state-wide population of over 6 million people, predominantly of Hausa-Fulani ethnicity, with Islam as the dominant religion (National Population Commission^24^. The State is largely rural, with most residents engaged in subsistence farming, animal rearing, and petty trading. Jigawa consistently records some of the poorest maternal and child health indicators in Nigeria, including high neonatal, infant, and under-five mortality rates^24,25^. The State has also reported high prevalence of diarrheal diseases among children under five^24^, which causes 24–30% of post-neonatal under-five deaths in Northern Nigeria^26^. This high burden is attributed to poor sanitation, unsafe water sources, and suboptimal infant feeding practices.

### Participant selection

Full methods for sampling and data collection have been published previously^27^. Our participants of interest were women aged 16–49 years who had reported breastfeeding in the previous two years. The ethnographic process evaluation was organised around six purposively selected health facility clusters from the main INSPIRING Jigawa trial, three from the intervention group and three from the control group, balanced for facility type (i.e., primary health centre, basic health centre, or health post). We then randomly selected five compounds in each cluster and recruited women aged 16–49 years with children younger than 5 years from these compounds using availability sampling, stratifying for age and wife position. Overall, 90 women were recruited into the wider ethnographic study.

Using purposive sampling, 36 women who reported active or recent breastfeeding within the preceding two years were selected from this cohort of women participating in the ethnography. The inclusion criterion of recent breastfeeding was to ensure the analysis focused on more recent experiences of infant feeding practices. To achieve balanced representation across study arms, we chose to select 18 women from the intervention clusters and 18 from the control clusters. This sample size was considered sufficient for in-depth qualitative analysis and was guided by the principle of thematic saturation; whereby no substantively new themes emerged with continued data collection. Participants ranged in age from 16 to 49 years and reflected the broader sociodemographic profile of rural Jigawa State, with most being married, having two to nine children, and having limited or no formal education.

### Data collection

In-depth life history interviews were conducted at three time points as part of the ethnographic design. These interviews involved extended, open-ended conversations that enabled participants to recount their experiences and narratives regarding infant feeding practices over time, including the sequence of decisions, challenges, and influences within their households. The interviews were complemented by monthly informal household visits, which captured everyday feeding practices, caregiving routines, and family interactions, and monthly observations at the six corresponding health facilities, which documented service delivery, counselling practices, and interactions between caregivers and health workers. This combined approach allowed for a rich, contextual understanding of infant feeding practices both at home and within the health system. For this paper, we used data from the midline interview conducted in July 2021, in addition to notes taken from monthly household and facility visits.

A semi-structured guide was used for the life-history interview, including questions focusing on feeding practices, and pattern of EBF within the wider household (Appendix 1). The guide was prepared by FS based on existing literature and formative field engagement and was discussed and refined with RAB, CK and AGF to ensure conceptual and contextual relevance. Interviews were conducted by three research assistants with prior qualitative experience, who received training on the study objectives, ethical conduct, and use of the interview guide. Training included guided discussions on the rephrasing of questions into Hausa language to ensure conceptual alignment amongst interviewers and cultural appropriateness. Interviews were conducted within each participant’s household at locations chosen by the participants to ensure privacy, comfort, and rapport. Each interview lasted approximately 60 min. At the end of each interview, participants were provided with a small, non-monetary incentive (washing detergent). This incentive was provided after participation and was not intended to influence recruitment or participant responses, in line with our ethical approvals.

All interviews were conducted in Hausa, audio-recorded with participant consent, and transcribed verbatim in Hausa before translation into English by the research assistants. To ensure translation accuracy and data quality, all transcripts were reviewed and cross-checked by FS against the original audio recordings. Any discrepancies were resolved through team discussion. Field notes from household and facility visits were typed immediately after each visit to preserve contextual detail. Several strategies were employed to enhance data quality and credibility, including prolonged engagement in the field, triangulation across interviews, household observations, and facility observations, routine debriefing meetings within the research team, and iterative review of emerging themes. Reflexive notes were maintained throughout data collection to document analytic decisions and researcher positionality.

### Analysis

Data was analysed by FS using reflexive thematic analysis^28^. Interview transcripts were read several times as a means of data familiarization and notes of initial trends in the data were taken; thoughts and feelings regarding the data and analytical process were also documented by FS. Following this, codes were generated inductively from the data to produce interpretive labels for pieces of information that were of importance to the research aim. These codes were iteratively refined through team discussions and comparison across transcripts, ensuring consistency and depth. Related codes were then grouped into broader themes by collapsing codes that shared similar concepts. To enhance triangulation and contextual validity, field notes from household and health facility visits were reviewed alongside the transcripts. The development of the initial coding framework was supported by RAB and AGF, while the organization of themes and sub-themes was discussed with CK prior to finalization. All authors provided feedback on the interpretation of the findings.

### Ethics

Ethical approval was obtained from Jigawa State Government (ref: JPHCDA/ADM/GEN/073/V. I) and the University College London Research Ethics Committee (ref: 3433/004). Approval was obtained from the Local Government, District, Ward and Village heads of every single community before data collection commenced in any of the communities. Verbal consent was sought from all participants, including informing them that participation in interviews was voluntary, data collected would be used for research purposes only and that they have the right to withdraw at any time.

## Results

Of the 90 women who participated in life-history interviews in the wider ethnographic study, a purposively selected sub-sample of 36 women included in this analysis were aged 16–49 years and had between 2 and 9 children.

Overall, we found women held positive attitudes towards breastfeeding, with some mothers reporting breastfeeding their children beyond two years, and the initiation of complementary feeding occurring at varying ages. Breastfeeding was widely regarded as an essential cultural norm, with other feeding practices—such as early water introduction, giving herbs, and offering water solutions like those infused with date palm—viewed as supplementary rather than alternatives to breastmilk. While some mothers adhered to the recommended six months of EBF, others introduced water as early as newborn period along with breastfeeding. Similarly, the timing of complementary food introduction lacked consistency, with mothers initiating CF between three and seven months, shaped by perceptions of infant readiness, and prevailing community norms. The reflexive thematic analysis generated two themes related to local definitions of feeding practices: (1)   Cognitive polyphasia in the definition of EBF; (2) perceptions of water as necessary and harmless for infant; and two themes related to how these norms and local contexts influence child nutrition (1) food insecurity and gender norms determine complementary feeding; (2) Structural barriers and misconceptions in the infant feeding–malnutrition pathway.

### Cognitive polyphasia in the definition of EBF

This theme captures the tension between mothers’ conceptual understanding of EBF and the contradictions evident in their infant feeding practices. Although most mothers could accurately articulate the global recommendation that EBF involves feeding an infant solely with breast milk from birth up to six months of age, without the addition of water, other liquids, or foods, their practical interpretations often diverged from this standard.

Many mothers demonstrated a form of cognitive polyphasia in their understanding of EBF - holding both biomedical and traditional beliefs simultaneously^26^. While they consistently avoided giving their infants other foods or formulas in line with EBF recommendations, they did not perceive giving water as a breach of EBF. One participant stated:

*“I never gave my babies milk or any baby formula apart from breast milk and water until they reach the stage of eating*

semi-solid and solid food like pap, rice, beans or tuwo” (P11, Intervention).

Another mother stated:

*“I only give them breast milk and water and when a child is about to start eating*,* he/she will display some signs” (P10*,* Intervention)*

These responses illustrate a partial and selective adherence to the principles of EBF, where mothers internalized parts of the guideline (excluding formula and foods) while disregarding others (excluding water). This was compounded by a lack of health literacy on why water should be avoided. One participant noted:

*“I just felt there was not going to be issues even if I give him water.” (P10*,* Control)*

Facility observations reinforced this finding. In one instance:

*“I engaged one of the women who came for routine immunization in discussion around exclusive breastfeeding. She told me she does practice but in addition with water. She claimed babies get thirsty. I am not surprise*d *to hear that*,* because they also tell us this in the communities. The In-charge stressed further on that as he said they can’t practice EBF without*.

*water and that is why diarrhoea among children is high*,* which eventually leads to malnutrition.” (Facility observation*,* facility 6)*

This account highlights how community members perceive water as an essential component of infant care, despite health education efforts promoting strict adherence to EBF. It also illustrates how health workers recognize the challenge of achieving EBF without water within this context, and link the prevailing practice to persistent child health problems like diarrhoea and malnutrition.

In some cases, mothers’ decisions to stop giving water were not driven by an understanding of medical recommendations but by personal or communal experiences of adverse events, such as a child choking. A participant recounted:

*“I once saw a woman who gave water to her baby and the baby got choked and almost fainted*,* and when the baby was taken to the hospital*,* the health workers advised that we should not give our babies water until after six months. For me*,* it was because of fear of what I saw happen to that baby that I stopped giving my children water until they reached six months.” (P 17*,* Intervention)*

These narratives and observations suggest that community’s practice of EBF is shaped by a combination of formal knowledge, personal interpretations, community norms, facility-level attitudes, and emotional responses to observed events — a dynamic that fosters cognitive polyphasia between what mothers know, believe, and practice.

### Perceptions of water as necessary and harmless for infants

Building on the first theme, women in the study commonly viewed water as exempt from the definition of exclusive breastfeeding. Water was perceived not only as harmless, but also as necessary for the health and wellbeing of infants. This belief gave mothers a degree of flexibility in their feeding practices and reflected the realities of competing priorities, practical constraints in daily life, and influences from other people’s experiences, as one mother explained:

*“I just felt there was not going to be issues even if I give him water.” (P10*, *Control)*

Some mothers reported modifying their infant feeding practices at night to suit personal convenience. One participant shared that she intentionally avoided breastfeeding at night because she believes that nighttime feeds encourage babies to develop habits that disturb their mothers’ sleep. This narrative highlights how personal and practical considerations, such as the desire for uninterrupted sleep, can lead to the early introduction of water. Mothers in this situation do not perceive water as a substitute for breastmilk but rather as a way to comfort or soothe a baby at night:

*“I don’t breastfeed at night because when a child gets used to breastfeeding at night*,* they become troublesome and wouldn’t allow me to sleep well. So*,* all my children are used to breastfeeding only from early morning to night time. I don’t wake up at midnight to breastfeed. If the baby cries*,* they may have water alone during that time.” (P17*,* Control)*

In other cases, past community experiences shaped this understanding that water is something desirable for infants. One respondent recalled:

*“So many years ago in this our compound*,* there was a child they started giving water*,* then after some days when they took him to collect his first vaccine at the hospital the mother was advised not to give the child water. That child cried throughout the whole day*,* even at night the child kept crying and when the mother decided to give the child water*,* he stopped crying and fell asleep. Since then*,* I never pick interest in practicing [exclusive breastfeeding].” (P13*,* Intervention)*

### Food insecurity and gender norms determine complementary feeding

Unlike EBF, there was limited understanding of adequate CF practices among mothers. Therefore, they exhibited considerable variation in both the timing of food introduction and the types of foods offered to infants and young children. While the WHO recommends introducing a range of complementary foods at six months of age alongside continued breastfeeding, we found flexible and inconsistent patterns, that were shaped by personal preferences, cultural norms, food insecurity, and limited nutrition awareness.

For example, mothers reported initiating CF from as early as three to five months to as late as seven months. Some mothers introduced light foods such as pap (a smooth and light-textured porridge made from grains such as maize, millet or sorghum) after five months, others extended breastfeeding with water up to seven months before introducing any complementary food, while a few women stated they adhered to the six-month EBF period.

*“I make pap for them to drink after about 5 months of birth then gradually*,* I teach them how to eat other foods like rice*,* beans*,* cassava and Tuwo.” (P4*,* Intervention)*

*“I breastfeed my children with breast milk and water for 7 months before I introduce light food like pap*,* and when they become older*,* I introduced other food to them.” (P3*,* Control)*

*“After 6 months I will start giving the child water*,* also I will start introducing pap to the child then later on solid food.” (P16*,* Control)*

The complementary foods introduced were predominantly locally available, cereal-based dishes with low dietary diversity. The most frequently mentioned first food was pap, followed by Tuwo (solid food made from maize, millet, sorghum) as the child grew older.

*“I do give them breast milk up to seven months then I will start introducing solid food to them like pap and Tuwo” (P13*,* I)*.

This mix of early, timely, and delayed initiation of CF, and lack of diversity, reflected factors such as maternal perceptions of child readiness, household food security, and socio-cultural expectations. Severe food insecurity was a key driver of limited dietary options, and many mothers described their reliance on staple foods, often prepared without oil, fish, or meat:

*“Here in our community*,* our only food is Tuwo and Kuka [green soup]. Sometimes we can’t even afford oil in the soup*,* not to talk of fish or meat. Such situation brings about malnutrition in children” (P4*,* Intervention).*

In addition to the testimonies from mothers, facility heads and nurses reported a concerning household dynamic in which husbands/men would go outside of the home to eat nutrient rich foods, while women and children who remain inside the home only had access to staples. This practice not only highlights gendered disparities in food access within households but the poor financial power of women, limiting their ability to procure diverse and nutrient-dense foods for themselves and their children, thereby exacerbating the challenges of achieving adequate CF.

*“They (facility head and nurses) claimed husbands/men in the community leave wives at home with one particular source of food (Tuwo) which is majorly carbohydrates while they go to tea joints to eat bread and egg or noodles and egg with tea*,* at times*,* they eat suya (roasted meat) too” (observational notes*,* Facility 1).*

Beyond economic hardship and unequal food distribution, participants also noted that children were frequently fed adult meals, without consideration for their specific nutritional needs:

*“Here*,* what I see them do is they give the children the same food we eat as adults but I don’t know if there is any other thing*,* they give them*,* and I also don’t think of any food that a child should be given” (P7*,* C).*

These accounts underscore the combined impact of poverty, intra-household food allocation dynamics, food insecurity, and limited knowledge about appropriate child feeding on how CF is practiced. There was limited awareness of adequate dietary diversity for child growth, and rather than a mis-aligned definition driving poor infant feeding practices, CF was reactive and reflective of circumstance.

### Structural barriers and misconceptions in the infant feeding–malnutrition pathway

Mothers’ accounts revealed a complex relationship between infant feeding practices, episodes of diarrhoea and malnutrition, but also hint at the underlying role of maternal nutrition. Many mothers described how inadequate breastfeeding, early or inappropriate CF, and recurrent childhood illnesses contributed to weight loss and poor growth in children. Diarrhoea was frequently mentioned as both a cause and a consequence of malnutrition. This is evident in the responses below:

*“The child that was malnourished in this household started with diarrhoea but they didn’t take her to hospital on time which led to malnutrition” (P3*,* C)*.

*“My son’s own started from teething fever then it led to diarrhoea and I noticed he started losing weight” (P16*,* C)*.

While mothers linked these feeding practices to poor health outcomes, they often lacked clear understanding of the underlying biomedical mechanisms by which poor feeding practices and unclean water increase the risk of diarrhoea and malnutrition. Their explanations were frequently shaped by cultural beliefs about hygiene, maternal status during breastfeeding, and childhood illnesses. For instance, some understood that when a breastfeeding mother becomes pregnant, it causes poor nutritional outcomes in the breastfeeding child:

*“The cause of malnutrition like I told you is dirt and when a woman is pregnant and she is still breastfeeding that makes the child to be malnourished”* (P6, Intervention).

*“The child was very small though it started with when the child was teething and he kept defecating which led to the child losing a lot of weight. Then I got pregnant while I was still breastfeeding the child and I didn’t wean the child*,* so he lost a lot of weight that led to him looking very slim; the hands and legs were thin*,* that’s all that happened” (P9*,* Control).*

Others emphasized the role of hygiene and timely healthcare seeking:

*“It is caused by dirtiness; you don’t wash your hands before you feed a child and you don’t take care of the kind of food you feed your children with*,* or a child is having diarrhoea and you won’t take the child to the hospital on time” (P17*,* Intervention)*.

Facility observations echoed these maternal beliefs. Informal discussions with health workers revealed that community members attributed malnutrition mainly to poor hygiene and environmental dirt, without properly considering the role of inadequate diet.

Facility observations also illustrated the scale of the malnutrition problem. In one facility observation, it was noted how shocking it was to observe the sheer number of malnourished children being brought in for care:

*“They came in masses. These (plumpy nuts) was given to more than 100 children” (observational notes*,* Facility 6).*

A facility staff, who was particularly open to engagement, discussed the high prevalence of malnutrition in the community and suggested health education interventions. However, he emphasized that such efforts would only achieve sustained impact if accompanied by| improvements in literacy, as low educational attainment significantly constrained health-seeking behaviors and feeding practices:

Another factor mentioned by facility doctor was community members literacy level which has great role in correcting both malnutrition and anaemia, he then caped it all with economic state of the country and increase in the price of food commodities (observational notes, Facility 1).

## Discussion

Our findings demonstrate a clear gap between mothers’ knowledge of EBF and their actual interpretation and practice within the community. Although most mothers could correctly define EBF as feeding an infant only breast milk for six months, it was common practice to introduce water as early as the newborn period, in addition to breastfeeding, driven by beliefs of water being both harmless and necessary. Conversely, adequate CF was not well understood, and practices were responsive to the context of food insecurity and challenges in access to nutrient-rich food given the pervasive poverty and catastrophic flooding events in this setting, making it difficult to follow EBF and CF recommendations.

Elsewhere, we have previously identified cultural and religious beliefs as primary reasons for early water introduction, such as the use of holy water for blessings or the perception that denying water is cruel^6^. In this deeper exploration, we noted that while there are symbolic reasons for giving water, the practice of giving water is supported by the belief that water is not harmful and that infants are seen to need water – both to quench thirst and to comfort the infant. These factors can interact to allow the practice to persist, despite the knowledge that water should not be given. Similar practices have been observed in other settings across Nigeria and Sub-Saharan Africa. Burba et al. (2025)^29^ reported that although a majority of mothers in northwestern Nigeria demonstrated good knowledge of EBF, actual practice was limited due to traditional beliefs about the necessity of water and herbal medicines for infants. Likewise, a study by Onah et al. (2014)^4^ from Nigeria found that awareness of EBF was high, but the prevalence of early supplementation with water and other fluids remained common. Abdullahi et al. (2017)^30^ also documented this knowledge-practice gap in Nigeria, attributing it to the persistence of cultural norms and family influences that override biomedical recommendations. However, our findings suggest that even mothers who are informed about EBF, perceive water as a negligible addition that doesn’t mean they are not practicing EBF.

Although caregivers widely believed that giving water to infants is harmless, this perception overlooks the potential health risks associated with unsafe drinking water. In settings like Jigawa—where several cholera outbreaks have occurred in recent years 2018, 2021, and most recently in 2024 with over 70 reported cases and associated deaths, predominantly among women and children^31–33^—introducing water to infants may increase vulnerability to waterborne diseases. Northern Nigeria also records one of the highest under-five mortality rates globally, with diarrhoeal illnesses accounting for roughly one in four of these deaths^25^. While participants did not explicitly link water introduction to diarrhoeal disease, the disconnect between the practice of giving water to infants and its potential health consequences highlights a critical gap in community understanding of why exclusive breastfeeding is recommended.

Similar misconceptions have been documented in other regions. For instance, in The Gambia, mothers believed that giving water or thin porridge was part of EBF, not recognizing that these additions could pose health risks^31^. In Ghana, health workers reported that some mothers perceived breastmilk as insufficient to quench infants’ thirst, leading to early water supplementation^32^. In the Democratic Republic of Congo, some mothers and grandmothers believed that water should be introduced to infants before six months, despite healthcare providers’ advice to the contrary^33^. These findings underscore the importance of not only promoting EBF but also ensuring that health education programs effectively communicate the reasons behind the WHO’s recommendations. Addressing misconceptions and providing clear explanations about the sufficiency of breast milk, alongside emphasizing the importance clean water and sanitation and adequate maternal nutrition, are crucial steps toward improving adherence to EBF and safeguarding infant health.

Patterns of CF reflected a reliance on locally available, low-diversity foods such as pap and Tuwo, introduced at varying ages. Inadequate dietary diversity and inappropriate CF practices – with both early and late introduction of foods posing issues, have been consistently associated with child undernutrition in Nigeria^34^ and other low-resource settings^35^. The socioeconomic realities of many households, including food insecurity and limited access to nutrient-rich foods, further constrained mothers’ ability to provide optimal CF, as also documented in a qualitative study in rural northern Nigeria^36^.

An important contribution of this study lies in showing how mothers made sense of the relationship between infant feeding practices and health outcomes such as diarrhoea and malnutrition. Their explanations often reflected culturally shaped interpretations—for example, attributing diarrhoea to teething or viewing malnutrition as a result of environmental dirt or maternal pregnancy during breastfeeding. Malnutrition in this community is influenced by multiple factors, including hygiene practices, birth spacing, maternal nutrition, infections, and feeding practices. However, ethnographic observations highlighted specific gaps in awareness within this community. While caregivers generally understood exclusive breastfeeding (EBF) to mean giving only breast milk, they lacked awareness of the dangers of introducing water during this period and of the appropriate timing and composition of complementary feeding (CF). These perceptions echo findings from earlier studies in northern Nigeria and other parts of Africa, where cultural beliefs strongly influence understandings of illness causation and child care practices^37,38^. Our findings highlight the dual burden of risk factors and culturally shaped beliefs in shaping infant feeding practices and child nutrition outcomes, where mothers contain multiple, seemingly contradictory knowledge systems together through their everyday practices. Suboptimal adherence to recommended breastfeeding and CF practices, alongside recurrent diarrheal episodes and delays in seeking care, contribute to a cycle of undernutrition, as outlined in global child survival frameworks^39^. Additionally, culturally embedded beliefs and practices, which hold significant meaning and value within the community, may influence feeding and healthcare behaviours in ways that can sometimes limit the adoption of evidence-based recommendations.

This study has several strengths, including a robust qualitative sample and prolonged ethnographic engagement, which allowed for in-depth insights into infant feeding practices within the community. The combination of life-history interviews and repeated household observations enabled triangulation between reported behaviours and everyday practices, strengthening the credibility of the findings.

We acknowledge several limitations. Participants’ responses may have been influenced by social desirability bias, particularly given the emphasis on exclusive breastfeeding within health services and the intervention context. This was mitigated through repeated household visits over an extended period, which helped build trust and rapport, and by triangulating interview data with observational field notes. Researcher positionality and reflexivity were also carefully considered. The lead researcher’s prior involvement in the INSPIRING project may have influenced data interpretation; this was addressed through the use of reflexive field notes, regular team discussions to interrogate emerging interpretations, and collaborative review of codes and themes with supervisors. Finally, while the findings provide rich contextual insights, they are specific to the study setting and should not be assumed to be directly generalisable to other contexts.

## Conclusion

This study identified the need for integrated, culturally sensitive health promotion interventions that address both the practical barriers to optimal feeding — such as food insecurity, at the same time as engaging with underlying beliefs about child health and nutrition. Community-based education programmes, delivered through trusted community structures such as women’s groups and religious leaders, could help to correct misconceptions about EBF, water introduction, and the causes of diarrhoea and malnutrition, while promoting affordable and context-appropriate CF practices.

## Supplementary Information

Below is the link to the electronic supplementary material.

Supplementary Material 1

Supplementary Material 2

## Data Availability

Data will be shared upon reasonable request.
