# Supplementary material for: “I just felt there was not going to be issues” exploring local definitions of exclusive breastfeeding and adequate complementary feeding within communities in Jigawa state, Nigeria
Source: Sci Rep. 2026 Feb 26;16:7741. doi: 10.1038/s41598-026-41749-z (PMC12948948; doi:10.1038/s41598-026-41749-z)
Supplement: Supplementary file 1 — Supplementary Material 1 [file 41598_2026_41749_MOESM1_ESM.docx]

**Supplementary Data 1: Interview topic guide**

**INSPIRING: Household life history and caregiver interview topic guide – 2^nd^**

**Note to interviewer:** The aim of this interview is to help create a longitudinal picture of perspectives and experiences of this family and their relationships and practices around pneumonia, child health and women’s empowerment. In This interview, the sections are shorter, and you should approach this interview in the style similar to your informal visits in previous months.

If participants provide one-word answers, remember that you can probe around each one for feelings, experiences and understandings about the issue (i.e., how did that make you feel? Why did it happen that way? etc).

**Section 1: Life history and background section**

**Interviewer introduction**: Thank you for agreeing to take part in these additional interviews. They will give us a chance to understand a bit more about your household, what is important to you, and how you go about your day-to-day activities to contribute to your family. We will ask you about a few different things.

1. **Can you tell me a bit about you?**

Have you spoken to or seen any of your family since we last spoke? (probe for relevant siblings, important family members, Aunts, etc).

How are things with your husband?

How are your children doing? are they still in school?

Who have you been spending time with recently? Who is the most special person in your life right now?

What are the two hardest things about your life right now?

What are the two best things about your life right now?

Can you tell me a story about something important that has happened in your family since we last spoke?

**Section 2: The household and wider community**

1. **Can you tell me a little bit about the rest of your household? (for each person, make sure you explore the prompt:** What causes the relationship to be this way?)

How has your relationship been with ….?

1. Your Husband’s other wives – and why? What causes the relationship to be this way?
2. Your Husband’s mother
3. Your Husband’s other family members
4. Your Husband’s other children
5. Any other people mentioned in question 2
6. Your children?
7. Was their any case of divorce since we last spoke? If yes, what led to that?
8. Has it been resolved? How was it resolved?
9. Would you like to tell/ share the story of another woman’s divorce if asked? Why/why not?
10. If no, who is in the best position to ask about that if that happens to one of the women we follow up?

Facilitator notes: don’t accept one-word answers like ‘good’ or ‘fine’. If you get a one-word answer, then you can ask them to tell you a story about a typical encounter with that family member.

Space for notes:

3. Who are the most important people in your community? Why?

Write in the space below who is named as important.

1. Since we last spoke, how are things with the family finances?

Prompts:

A) Who is employed? Does anyone have a new job? Did someone lose a job? Did crops fail, did herds fall ill/die? Anything that has impacted the family finances is relevant

B) Have things changed with how your family shares money in the household? If yes, what and how?

C) Have things changed with how your family organises and spends money for:

i) food - If yes, what and how?

ii) health - If yes, what and how?

iii) education? - If yes, what and how?

D) Has your role in family finances changed since we last spoke?

E) How does your family gain access to food when money is low? What do you do when there is not enough food available in your household?

1. **Tell me about your religion and tradition?**

Note to interviewer: If the participant answers any of the below in their initial answer, please do not repeat the question.

- - 1. How does your religion help you with what you do every day?
    2. Can you tell me about a recent challenge in your life that your religion has helped you with?

**Care seeking experiences and use of health services**

1. In the questionnaire you completed recently, you were asked some questions about a time that your child was sick. Could you tell me a story about one of those times that has happened since we first spoke? How did you first notice your child was ill? (note to interviewer – this should be a different episode from what was previously discussed)

*Prompts:*

What symptoms did you notice?

When did you decide to seek care?

How did you make that decision?

Where did you decide to go?

How do other people in your household contribute to your decision?

1. Have you visited the health services in the community since we last spoke? Can you tell me a story about a recent time that you accessed services and what happened?

**Interviewer note: please prompt around the story told, to understand who, what, where, when and why of the experience.**

**Mental and emotional wellbeing (linked to WEMWBS-7)**

1. In the questionnaire you completed recently, you were asked some questions about a time when you felt like you were dealing with problems well. Can you give me an example of a story or time in your life when you felt this way since we last spoke?
   1. Is this a new thing to happen, in your life? How has it changed? Why has it changed?

**Interviewer note: please prompt around the story told, to understand who, what, where, when and why of the experience.**

1. The questionnaire also asked about at time in your life when you were feeling optimistic about the future. Can you give me an example of a story or time in your life when you felt this way since we last spoke?

- Is this a new thing to happen, in your life? How has it changed? Why has it changed?

**Interviewer note: please prompt around the story told, to understand who, what, where, when and why of the experience.**

1. The questionnaire also asked about a time in your life where you felt close to other people. Can you give me an example of a story or time in your life where you felt this way since we last spoke?
   1. Is this a new thing to happen, in your life? How has it changed? Why has it changed?

**Knowledge about child health issues**

1. What do you think is the biggest health challenge that children encounter?
   1. Has this changed since we last spoke? Why/why not?
2. What do you do to keep your children healthy?
   1. Has this changed since we last spoke? What is different? Why/why not
3. Have you heard about a health issue called pneumonia?

*If yes:*

- - 1. Can you describe it to me? What does it look like?
    2. How do you know a child has it?

Prompt: What are the symptoms? What would the child look like?

- 1. What is the best way to treat it?
     1. Who taught you how to do this?

Prompt: Did your mother teach you that? Is it a health care provider? Other family members? Is that how it is always done?

*If no:*

Interviewer: Describe a child’s symptoms that link to pneumonia, and ask the woman what they would do to treat that child.

**Knowledge, perception and practice of Exclusive Breastfeeding**

Can you tell me a little bit about how you feed/fed your children?

1. How did you decide to breastfeed? Who made the decision in your household?

Probe for why that decision was made by that person, and how the participant felt about that.

1. What was breastfeeding like for you?

Have you heard about Exclusive breastfeeding? What does that term mean to you?

1. What do know about exclusive breastfeeding? What is exclusive breastfeeding?
2. How long should a child be exclusively fed?
3. Did you practice EBF with your last child? Why/why not
4. What are the positives of exclusive breastfeeding to you?
5. What things make exclusive breastfeeding difficult for you?
6. Who determines whether a child would be exclusively breastfed? Give reasons please
7. What influence do members of your household have in the practice of EBF?
8. How many times does a baby needs to breastfeed in a day?

**Knowledge on malnutrition**

1. What is malnutrition?
2. How do you know a child is malnourished?
3. What are the likely causes of malnutrition?
4. How can that be prevented/treated?
5. Who is the decision maker in your family?
6. Who makes decision about when to have children?
7. Why is that so?

**Knowledge and experience of the intervention**

Note to interviewer: Please only ask this question for the families that are in intervention clusters. DO NOT ASK IF THE FAMILY IS IN A CONTROL CLUSTER.

1. In your community, women’s and men’s group have been started as a way to improve knowledge around pneumonia and other health issues that children face.

Have you heard about these groups? If yes:

- 1. Can you tell me what you have heard? What do others in the community say about these groups?
  2. What do you think about these groups? How are they important? How are they helpful?
  3. What does your family think about these groups?
  4. Have you participated in any of these groups? **If yes:**
     1. What has your experience of the groups been?
     2. What does your group work on?
     3. How did you join the group? Who made the decision?
     4. Who often make decision on whether women can join a group or not? Why is that so?
     5. What would you say about other women’s interest to join the group?
     6. How has your time in the groups affected you?
  5. Is anyone else in your family a member of these groups?

IF the participant says they have NOT joined a group:

- 1. Why did you decide not to join a group?
  2. Who made the decision? Would you have made a different decision if given the chance?
  3. Could anything have helped you decide to join the group?

**Knowledge on the practice of COVID-19**

1. What do you know about COVID-19?
2. do you believe in its existence? Why/why not
3. What are the symptoms?
4. How can it be prevented?
5. Have you heard about vaccines for COVID-19? Tell me about what you have heard.
   1. Probe for different stories about where it came from, what vaccines are made of, who makes them, what the side effects are.
   2. Where did you hear this information?
   3. Do you trust this information? Why?
6. If the vaccine is brought to your facility, will you be willing to get? Why/why not

Closing question:

Is there anything else you would like to say, or talk about with me today?
