# Supplementary material for: “I just felt there was not going to be issues” exploring local definitions of exclusive breastfeeding and adequate complementary feeding within communities in Jigawa state, Nigeria
Source: Sci Rep. 2026 Feb 26;16:7741. doi: 10.1038/s41598-026-41749-z (PMC12948948; doi:10.1038/s41598-026-41749-z)
Supplement: Supplementary file 2 — Supplementary Material 2 [file 41598_2026_41749_MOESM2_ESM.docx]

Supplementary File 1: COREQ checklist

Consolidated criteria for reporting qualitative studies (COREQ): 32-item checklist

Developed from:

Tong A, Sainsbury P, Craig J. Consolidated criteria for reporting qualitative research (COREQ): a 32-item checklist for interviews and focus groups. International Journal for Quality in Health Care. 2007. Volume 19, Number 6: pp. 349 – 357

| **Item No** | | **Guide Questions/Description** | **Reported on Page #** |  |
| --- | --- | --- | --- | --- |
| **Domain 1: Research team and reflexivity** | | | |  |
| **Personal Characteristics** | | | |  |
| 1. Interviewer/ facilitator | | Which author/s conducted the interview or focus group?  *The interviews were conducted by the lead researcher (the first author) and three trained research assistants.* | Pg 6 |  |
| 2. Credentials | | What were the researcher’s credentials? E.g., PhD, MD  *The lead researcher was a doctoral candidate in maternal and child health, and the research assistants had prior training in qualitative data collection* | Pg 6 |  |
| 3. Occupation | | What was their occupation at the time of the study?  *The lead researcher was a doctoral student, supported by three trained research assistants.* | Pg 6 |  |
| 4. Gender | | Was the researcher male or female?  *Female (FS)* | Pg 6 |  |
| 5. Experience and training | | What experience or training did the researcher have?  *The lead researcher had prior training in qualitative methods and ethnography and experience conducting field research in maternal and child health. The research assistants were trained in qualitative data collection and interview techniques before data collection.* | Pg 6 |  |
| **Relationship with participants** | | | |  |
| 6. Relationship established | | Was a relationship established prior to study commencement?  *The lead researcher and research assistants did not have a prior relationship with participants. Relationships and trust were built during recruitment and early interactions, facilitated by local community leaders.* | Pg 7 |  |
| 7. Participant knowledge of the interviewer | | What did the participants know about the researcher? e.g. personal goals, reasons for doing the research?  *Participants were informed that the study focused on infant feeding and child nutrition practices, that the lead researcher was affiliated with a university, and that participation was voluntary and confidential* | Pg 7 |  |
| 8. Interviewer characteristics | | What characteristics were reported about the interviewer/facilitator? e.g. Bias, assumptions, reasons and interests in the research topic  *The lead researcher’s prior involvement in the INSPIRING project may have influenced data interpretation. This was addressed through reflexive field notes, regular team discussions to interrogate emerging interpretations, and collaborative review of codes and themes with supervisors.* | Pg 16 |  |
| **Domain 2: study design** | | |  |  |
| **Theoretical framework** | | |  |  |
| 9. Methodological orientation and Theory | What methodological orientation was stated to underpin the study? e.g. grounded theory, discourse analysis, ethnography, phenomenology, content analysis  *The study used an ethnographic and life-history approach, guided by reflexive thematic analysis to explore infant feeding practices and the sociocultural context of child nutrition.* | Pg 4 |  |  |
| **Participant selection** | | |  |  |
| 10. Sampling | How were participants selected? e.g., purposive, convenience, consecutive, snowball  *Participants were purposively selected from the ethnographic cohort to ensure variation in age, parity, and child feeding experiences. A total of 90 women completed life-history interviews, of which 36 were selected for detailed analysis* | Pg 5 |  |  |
| 11. Method of approach | How were participants approached? e.g., face-to-face, telephone, mail, email  *Participants were recruited in person, with introductions facilitated by community leaders to explain the study purpose and obtain consent.* | Pg 7 |  |  |
| 12. Sample size | How many participants were in the study?  *36 participants were included in this analysis from a larger ethnographic cohort of 90 women who completed life-history interviews.* | Pg 5 |  |  |
| 13. Non-participation Setting | How many people refused to participate or dropped out? Reasons?  *All approached participants consented to participate. No one refused or dropped out* | N/A |  |  |
| 14. Setting of data collection | Where was the data collected? e.g., home, clinic, workplace  *Life-history interviews were conducted in participants’ homes including observations to ensure comfort and privacy. Facility observations were conducted in local health facilities as part of the ethnographic study.* | Pg 6 |  |  |
| 15. Presence of nonparticipants | Was anyone else present besides the participants and researchers?  *Only the participant and the interviewer were present during interviews*. | N/A |  |  |
| 16. Description of sample | What are the important characteristics of the sample? e.g. demographic data, date  *The study included women of childbearing age (16–49 years), with 2–9 children, primarily married and engaged in home-based or nearby income-generating activities (e.g., petty trading, knitting, small-scale farming). Participants were part of a larger ethnographic cohort and were selected from Kiyawa LGA, Jigawa State, Nigeria. Data were collected between July 2020 and November 2022 as part of the INSPIRING Jigawa cluster randomized controlled trial process evaluation (ISRCTN3921355).* | Pg 4 |  |  |
| **Data collection** | | |  | No |
| 17. Interview guide | Were questions, prompts, and guides provided by the authors? Was it pilot tested?  *A semi-structured guide was used for the life-history interviews, including questions on infant feeding practices and patterns of exclusive breastfeeding within the household (Appendix 1). The guide was prepared by FS based on existing literature and formative field engagement, and discussed and refined with RAB, CK, and AGF to ensure conceptual and contextual relevance.* | Pg 6 |  |  |
| 18. Repeat interviews | Were repeat interviews carried out? If yes, how many?  *Not applicable; each participant was interviewed once.* | N/A |  |  |
| 19. Audio/visual recording | Did the research use audio or visual recording to collect the data?  *All life-history interviews were audio-recorded with participant consent* | Pg.6 |  |  |
| 20. Field notes | Were field notes made during and/or after the interview or focus group?  *Field notes from household and facility visits were taken during and immediately after each session and typed promptly to preserve contextual detail. Reflexive journals were maintained throughout data collection to document observations and support analysis.* | Pg.6, Pg 7 |  |  |
| 21. Duration | What was the duration of the interviews or focus group?  *Each life-history interview lasted approximately 60 minutes*. | Pg 6 |  |  |
| 22. Data saturation | Was data saturation discussed?  *Data collection continued until thematic saturation was reached within the ethnographic cohort. Saturation was assessed iteratively during analysis when no new themes or insights were emerging from additional interviews* | Pg 5 |  |  |
| 23. Transcripts returned | Were transcripts returned to participants for comment and/or correction?  *Transcripts were not returned to participants for comment or correction* | N/A |  |  |
| **Domain 3: analysis and findings** | | |  |  |
| **Data analysis** | | |  |  |
| 24. Number of data coders | How many data coders coded the data?  *The primary coding was conducted by the lead researcher (FS). Codes and emerging themes were iteratively reviewed and refined through collaborative discussions with three team members (RAB, AGF, and CK) to ensure consistency and depth.* | Pg 7 |  |  |
| 25. Description of the coding tree | Did the authors provide a description of the coding tree?  *The coding process and structure are described in detail: transcripts were read multiple times for familiarization, initial codes were generated inductively, iteratively refined through team discussions, and then grouped into broader themes. Related codes sharing similar concepts were collapsed to form sub-themes, with field notes reviewed alongside transcripts to enhance contextual validity. The initial coding framework was developed with support from RAB and AGF, and theme organization was finalized with CK.* | Pg 7 |  |  |
| 26. Derivation of themes | Were themes identified in advance or derived from the data?  *Themes were derived inductively from the data through reflexive thematic analysis. Codes were generated from the transcripts without pre-defined categories, and related codes were grouped iteratively into broader themes and sub-themes during team discussions.* | Pg 7 |  |  |
| 27. Software | What software, if applicable, was used to manage the data?  *No specialized qualitative data analysis software was used. Coding and thematic analysis were conducted manually using Microsoft Word and Excel to organize and manage codes and themes.* | Pg 7 |  |  |
| 28. Participant checking | Did participants provide feedback on the findings?  *Participants did not provide formal feedback on the findings. Key summaries and clarifications were discussed informally with participants during follow-up when necessary.* | N/A |  |  |
| **Reporting** | | |  |  |
| 29. Quotations presented | Were participant quotations presented to illustrate the themes/findings? Was each quotation identified? e.g., participant number  *Participant quotations were included to illustrate the themes and findings. Each quotation is identified using a participant code (e.g., P1, P2, F1) to maintain confidentiality and allow traceability to the source.* | Pg 10-14 |  |  |
| 30. Data and findings consistent | Was there consistency between the data presented and the findings?  *Yes, the findings are fully supported by the data. Participant quotations, field notes, and observational data consistently align with the themes presented, demonstrating coherence between the raw data and the interpretations.* | Pg 10-14 |  |  |
| 31. Clarity of major themes | Were major themes clearly presented in the findings?  *Yes, the major themes were clearly presented in the findings. Each theme is described with supporting participant quotations and contextual explanations to ensure clarity and coherence.* | Pg 10-14 |  |  |
| 32. Clarity of minor themes | Is there a description of diverse cases or a discussion of minor themes?  *Yes, the analysis includes discussion of diverse cases and minor themes. Variations in participant experiences, including differences in household context, maternal age, parity, and feeding practices, are described alongside the major themes to provide a nuanced understanding of infant feeding and child nutrition practices.* | Pg 10-14 |  |  |
